# A realist approach to eliciting the initial programme theory of the antiretroviral treatment adherence club intervention in the Western Cape Province, South Africa

**DOI:** 10.1186/s12874-018-0503-0

**Published:** 2018-05-25

**Authors:** Ferdinand C. Mukumbang, Bruno Marchal, Sara Van Belle, Brian van Wyk

**Affiliations:** 1 School of Public Health, University of the Western Cape, Cape town, South Africa; 2 Department of Public Health, Institute of Tropical Medicine, Antwerp, Belgium

**Keywords:** Adherence, Adherence club., Antiretroviral therapy., Configurational mapping., Intervention-Context-Actor-mechanism-outcome configuration., Generative mechanisms., Programme theory., Realist evaluation., Retention in care., Retroduction.

## Abstract

**Background:**

The successful initiation of people living with HIV/AIDS on antiretroviral therapy (ART) in South Africa has engendered challenges of poor retention in care and suboptimal adherence to medication. The adherence club intervention was implemented in the Metropolitan area of the Western Cape Province to address these challenges. The adherence club programme has shown potential to relieve clinic congestion, improve retention in care and enhance treatment adherence in the context of rapidly growing HIV patient populations being initiated on ART. Nevertheless, how and why the adherence club intervention works is not clearly understood. We aimed to elicit an initial programme theory as the first phase of the realist evaluation of the adherence club intervention in the Western Cape Province.

**Methods:**

The realist evaluation approach guided the elicitation study. First, information was obtained from an exploratory qualitative study of programme designers’ and managers’ assumptions of the intervention. Second, a document review of the design, rollout, implementation and outcome of the adherence clubs followed. Third, a systematic review of available studies on group-based ART adherence support models in Sub-Saharan Africa was done, and finally, a scoping review of social, cognitive and behavioural theories that have been applied to explain adherence to ART. We used the realist evaluation heuristic tool (Intervention-context-actors-mechanism-outcome) to synthesise information from the sources into a configurational map. The configurational mapping, alignment of a specific combination of attributes, was based on the generative causality logic – retroduction.

**Results:**

We identified two alternative theories: The first theory supposes that patients become encouraged, empowered and motivated, through the adherence club intervention to remain in care and adhere to the treatment. The second theory suggests that stable patients on ART are being nudged through club rules and regulations to remain in care and adhere to the treatment with the goal to decongest the primary health care facilities.

**Conclusion:**

The initial programme theory describes how (dynamics) and why (theories) the adherence club intervention is expected to work. By testing theories in “real intervention cases” using the realist evaluation approach, the theories can be modified, refuted and/or reconstructed to elicit a refined theory of how and why the adherence club intervention works.

**Electronic supplementary material:**

The online version of this article (10.1186/s12874-018-0503-0) contains supplementary material, which is available to authorized users.

## Background

By 2017, an estimated 37 million people were living with HIV and AIDS (PLWHA) globally and as of June 2017, 20.9 million PLWHA were accessing antiretroviral therapy (ART) [1]. Nevertheless, more than one third of patients are not adhering to treatment - an estimated 62% of PLWHA are adhering to their ART (taking ≥90% of ART) worldwide [2]. It has been reported that adherence to ART is better in sub-Saharan Africa compared to North America (77% versus 55%) [3].

In 2016, it was estimated that one in every seven (14%) people in South Africa were living with HIV/AIDS [4]. With an estimated 7 million PLWHA, South Africa has the highest number of PLWHA in the world [5]. South Africa, in 2011, had a 75% increase in access to ART, becoming the largest ART programme in the world with an estimated 3.3 million PLWHA currently initiated on ART [6]. Managing a large number of patients within a large ART programme poses various challenges. Prominent among these challenges are the problems of sub-optimal retention in ART care (high levels of lost-to-follow-up), poor adherence to medication and overcrowded health care facilities [7].

The impact of the growing numbers of patients in care was demonstrated by Médecins Sans Frontières (MSF) at the Ubuntu Clinic, a public health clinic in a densely populated, low-income residential area in Cape Town. This site was the largest ART clinic in the Cape Town Metro District, situated in an area with an extremely high HIV prevalence [8]. From 2006, the capacity of the facility to enrol new patients on ART showed a decline*.* As the clinic became saturated, the loss-to-follow-up rates of patients 12 months after enrolment increased with each successive annual cohort initiated on ART from 2005 to 2008. The decline was largely attributed to the facility-based and staff intensive model of care that was used for the management of PLWHA on ART [9]. The context of scarcity of nurses and doctors, accentuates the need for a more efficient model for managing large cohorts of patients on ART and specifically with the effective use of community-based strategies.

In the search for effective long-term retention models, the adherence club intervention, a differentiated care model [10] – consisting of streamlined HIV treatment and care adapted to the needs of a targeted group – was developed and piloted at Ubuntu Clinic. The adherence club is comprised of a group of patients whose appointments have been harmonised. Patients attend sessions that are modular and that can theoretically be placed outside of the clinic to reduce further congestion.

While the original conceptualisation of the adherence club intervention was facility-based (conducted within the premises of the facility) [11], community-based [12] and home-based [13] (out-of-clinic adherence club models) have also been piloted and implemented to further decentralise ART services. The goal of the out-of-clinic versions of the adherence clubs is to remove important health system barriers to retention in care and medication adherence such as long distances to clinic. Our study focused on the facility-based adherence club intervention.

The adherence club model demonstrated promising outcomes in terms of improved patient flow, an increase in the monthly enrolment of patients on ART and decreased loss-to-follow-up while increasing the overall number of patients in care [8]. Two years after the first enrolment of patients in the adherence clubs, only 2.4% of club patients had a negative outcome – 0.7% had died and 1.7% were lost to follow-up [11]. Altogether, 97.6% of patients were still in care: 89.5% remained in the club system, 4.8% had returned to mainstream care at Ubuntu clinic, and 3.3% had been transferred to other clinics [11].

Based on these results, the adherence club model was selected as a potential intervention to address the challenges of poor patient retention in care, suboptimal adherence to ART and health care facility congestion [14]. In 2011, the model was rolled out as a system improvement intervention aiming at streamlining the treatment and care of ‘stable’ patients in the Western Cape Province of South Africa. The rollout and implementation of the adherence club model was conceived and executed through the collaboration between the Western Cape Provincial Department of Health (WC DoH), the non-governmental organisation Treatment Action Campaign (TAC), the Cape Town Municipality City Health department (CoCT DoH), MSF, and the Institute for Healthcare Improvement (IHI).

**Fig. 1:**
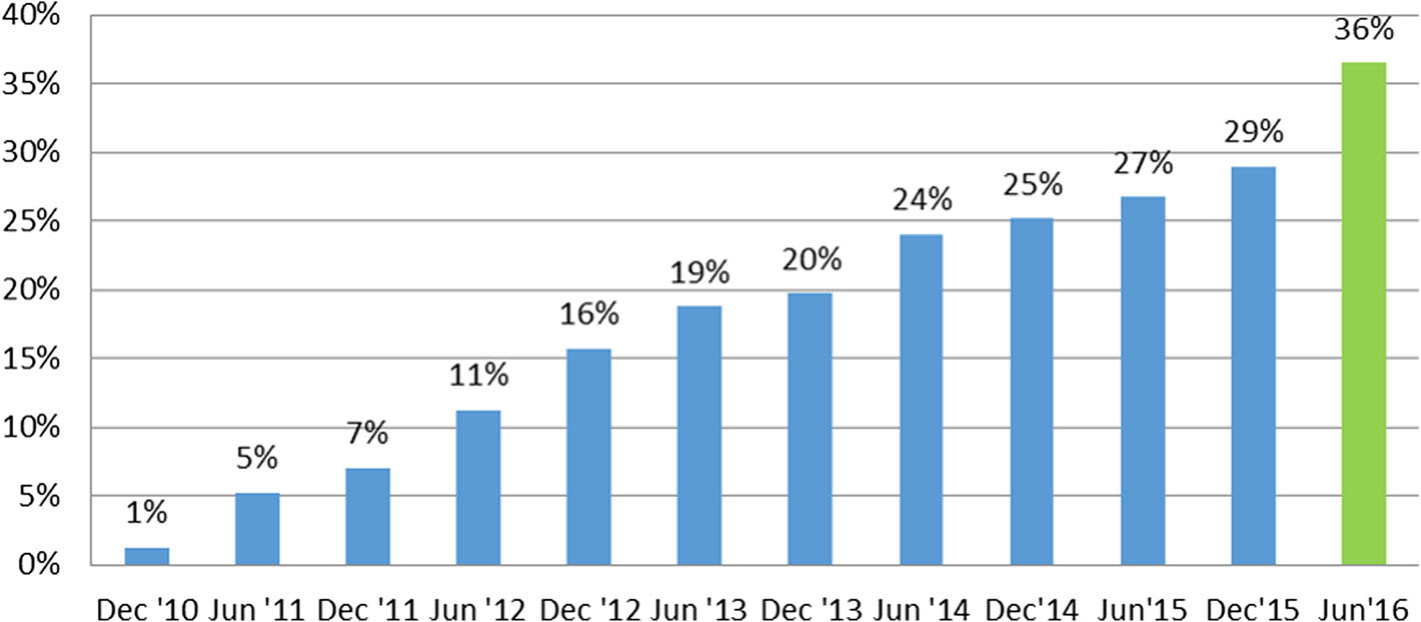
Percentage of patients in adherence clubs in the Cape Metropolitan Region

During the first phase of the rollout, from January 2011 to March 2015, 77% of ART sites in the Cape Metro area of the Western Cape Province implemented the adherence club intervention [14]. The graph (Fig. 1) below shows the progressive coverage of patients on ART in the Western Cape Province by the adherence club care model from December 2010 to June 2016.

This article reports on the results of the first phase of a study called “A realist evaluation of the antiretroviral treatment adherence club programme in selected primary health care facilities in the metropolitan area of Western Cape Province, South Africa” [15]. While other papers describe how the adherence club is organised and executed [12, 13, 16–18], we provide, in this paper, a comprehensive description of the programme based on a document review and participant observations of adherence club sessions in six primary health care facilities. Furthermore, we present the initial programme theory of the adherence club intervention based on the realist logic – i.e. exploring how, why, for whom and in what circumstances the adherence club intervention is *expected* to work.

### Methodological approach

#### Realist evaluation

Realist evaluation seeks to understand how and why, for whom, and under what circumstances a programme works (or not) [19, 20]. The philosophical basis of realist evaluation is scientific realism [19]. The realist evaluator aims to identify the context-mechanism-outcome (CMO) causal relationship in order to explain “how, why, for whom and under what conditions a programme works” [21]. Following the understanding that people are not passive recipients of innovations [22] and that programmes can only work when the relevant actors adopt either all or parts of the intervention modalities, Van Belle [23] and Mukumbang et al. [24] elaborated on the CMO configurational logic proposed by Pawson and Tilley [19] to include components of the ‘Intervention’ and the ‘Actors’. These authors suggested that an intervention-context-actor-mechanism-outcome (ICAMO) configuration would provide a better analytical tool because aspects of the intervention, which provide the mechanisms and the actors through whom the intervention works are accounted for. Thus, in this article, we adopted the ICAMO heuristic tool.

Because realist evaluation is focused on providing explanatory models, it has the potential to open the “black box” of programmes by making explicit the generative mechanisms to explain how the programme modalities lead to the intended outcome(s) [25]. For this reason, realist evaluation is recommended as an appropriate approach for capturing the complexities of health care interventions during their evaluations [26, 27].

The realist methodology is a ‘theory driven, interpretative approach to uncovering underlying middle-range theories (or logics) driving interventions and their multiple components, as well as illuminating the contextual factors that influence mechanisms of change to produce outcomes’ [28]. The interpretive approach is driven by various forms of reasoning; deductive methods (based on testing specific hypothesis) and inductive reasoning (formulating general inferences), but central to the realist method of inquiry is abductive or retroductive reasoning [29]. Retroduction is a mode of inference in which events are explained by postulating (and identifying) mechanisms which are capable of producing outcomes [30]. According to Wynn and Williams [31], retroduction is characterised by the use of causal mechanisms as the basis for explanation, the possibility for multiple potential explanations, and the understanding that these causal mechanisms may or may not be observable empirically.

Typically, realist evaluations start with an initial programme theory (hypothesis) and end with a more refined theory. Therefore, the evaluator hypothesises in advance the intervention (I) (or its components), the relevant actors (A), mechanisms that are likely to operate (M), the contexts in which they might operate (C) and the outcomes that will be observed if they operate as expected (O). This hypothesis is formulated by conceptualising the components of a programme implementation process (programme modalities, context, actors, mechanisms and outcomes) to form theories about the underlying causes to arrive at explanations of what we observe. Based on this, realist evaluators seek to understand how and why programmes work by formulating programme theories.

According to Westhorp [32], *“*Realist evaluation is most appropriate for evaluating new initiatives or programmes that seem to work but where ‘how and for whom’ is not yet understood; programmes that have previously demonstrated mixed patterns of outcomes; and those that will be scaled up, to understand how to adapt the intervention to new contexts.” Since the adherence club intervention fulfils all the above conditions, we adopted the realist evaluation approach for the evaluation of the adherence club intervention.

#### The Programme theory

One of the central elements of realist evaluation is the programme theory. Realist evaluation starts and ends with a theory or with theories. Thus, eliciting an initial programme theory is a “pre-requisite of realist evaluation” methodology [32]. Developing a programme theory follows from the notion that programmes are theory-incarnate [19]. A programme theory is described as “a set of explicit or implicit assumptions of how the programme should be organised and why the programme is expected to work” [33].

Programme theories link activities and outcomes to explain how and why the desired change is expected to take place and represent how “the mechanisms introduced by the programme into pre-existing contexts can generate outcomes” [34]. This process is guided by a ‘generative’ model of causality, in which causal links are demonstrated through a fine-grained explanation of what happens between cause and effect.

Developing programme theories serves two main purposes: as a planning tool and/or as an evaluation tool. If used for planning, Sharpe [35] suggests that it is beneficial to develop a programme theory prior to the start of the programme. In this instance, the programme theory indicates how different elements of the programme are intended to work together and to identify the intermediate outcomes of a programme or an intervention [36]. This gives a clear indication of the goals and objectives of the programme and of the pathways through which they could be attained.

Programme theories are also used to guide monitoring and evaluation [36]. They are especially important for the evaluation of complicated and complex aspects of programmes [37]. To this end, the goal of the evaluator(s) is to understand not only patterns related to the outcome of the intervention but also to reveal how and why the intervention attains the outcome of interest [38]. It is noteworthy that the evaluation of the programme’s theory is an evaluation of the programme rather than the evaluation of the programme theory [35]. Whatever their use, programme theories should be concrete enough to be tested and refined through empirical research, and abstract enough to generalise from the case-specific theories [39].

What differentiates the programme theory in realist evaluation from programme theories as conceived in other theory-driven approaches, such as Theory of Change [40] or Theory-driven evaluation [41, 42], is that realist evaluation specifies what mechanisms will generate the outcomes in what context. Thus, a realist programme theory provides a conceptual framework for putting the underlying CMO components centre stage [43].

## Methods

To describe the state-of-the-art adherence club intervention, we adopted a descriptive research approach. We carried out a document review of 12 programme documents describing the adherence club model and implementation strategy, the adherence club toolkits, policy documents, implementation reports and online-news (Additional file 1). To obtain these relevant documents, the first author searched various databases (PubMed, Google search, Google Scholar, and EBSCOhost) and relevant websites (MSF, WC DoH and Health E-news) using the terms “adherence club”, “ART adherence club”, “ART clubs”, “facility-based adherence club”, and “MSF innovation in ART management in South Africa” [44]. In addition, we conducted 24 structured nonparticipant observations of the adherence club programme in six facilities in the Western Cape Province – three with a record of good retention in care rates (≥ 80%) and three with a record of poor adherence to care rates (≤ 70%) in 2014 (ART retention in care report 2015). Nonparticipant observations allowed us to gain insights into the various types of club sessions, different activities and the dynamics of interactions between the patients with each another and with the health care providers. We used an observation guide that details the interactions, processes, or behaviours to be observed during the club sessions (Additional file 2).

Regarding eliciting the initial programme theory of the adherence club intervention, we adopted an elicitation research approach [45] – an approach that employs any number of data collection techniques to gather information (information gleaning). Eliciting the programme theory or theories of a programme in the realist sense entails identifying and making explicit the elements of the intervention, actors, mechanisms, outcomes, and contexts using the concept of generative causality.

**Fig. 2:**
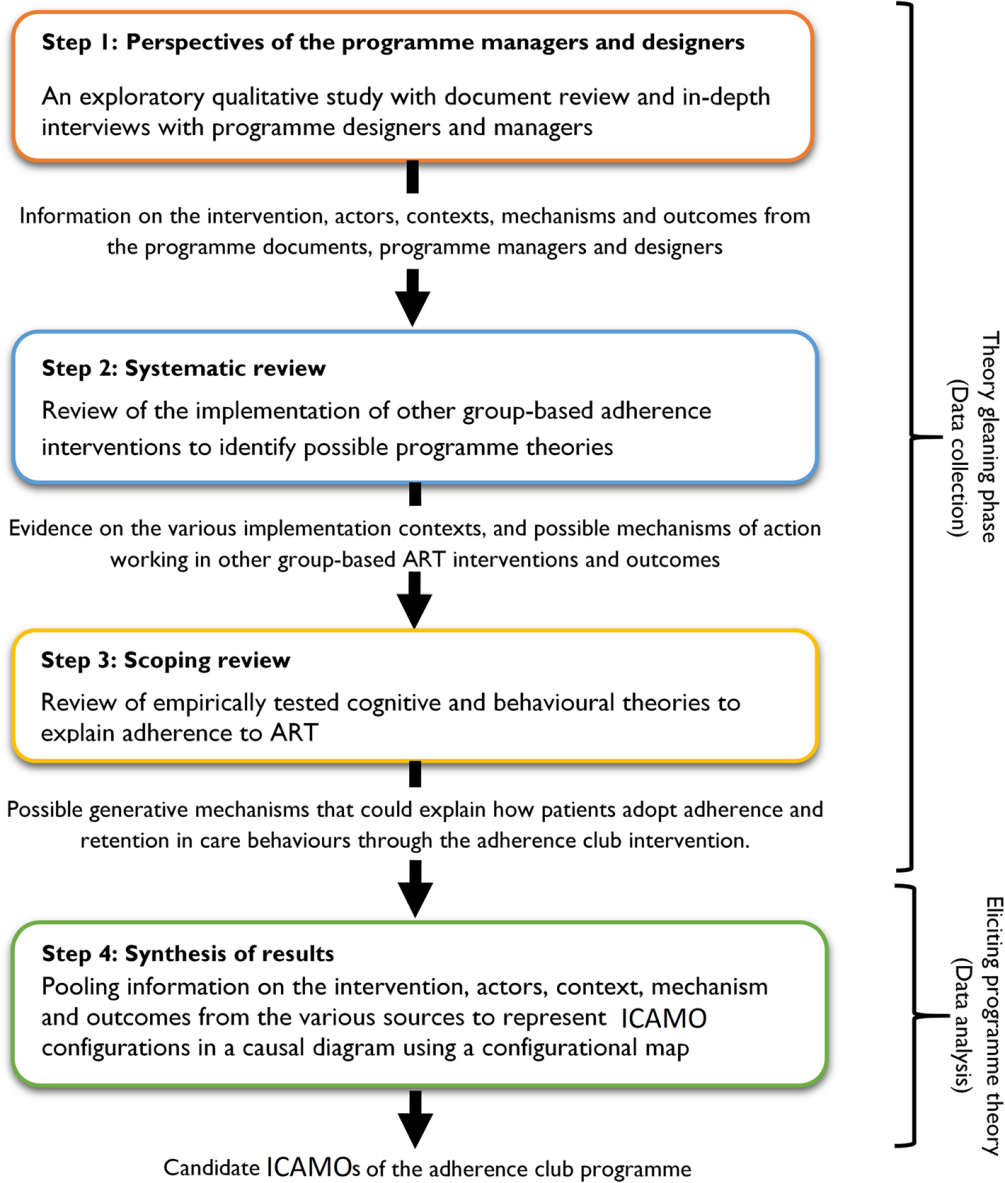
Steps employed to elicit the final Intervention-Context-Actor-Mechanism-Outcome configuration hypothesis

To obtain information on the relevant intervention, actors, mechanisms, outcomes and contexts, realist evaluators typically review programme documentation (policy documents, implementation reports, programme descriptions, etc.), interview or discuss with various programme stakeholders, and/or draw on a systematic review of previous research and evaluation literature. Then, these elements are connected and aligned using the ICAMO configurational tool following the generative model to obtain the programme theory or theories (theory gleaning). Figure 2 shows the various methods applied to obtain relevant information for the formation of the initial programme theory of the adherence club intervention. The theory gleaning process is described in more details in the results section.

### Data collection (information gleaning)

**Fig. 3:**
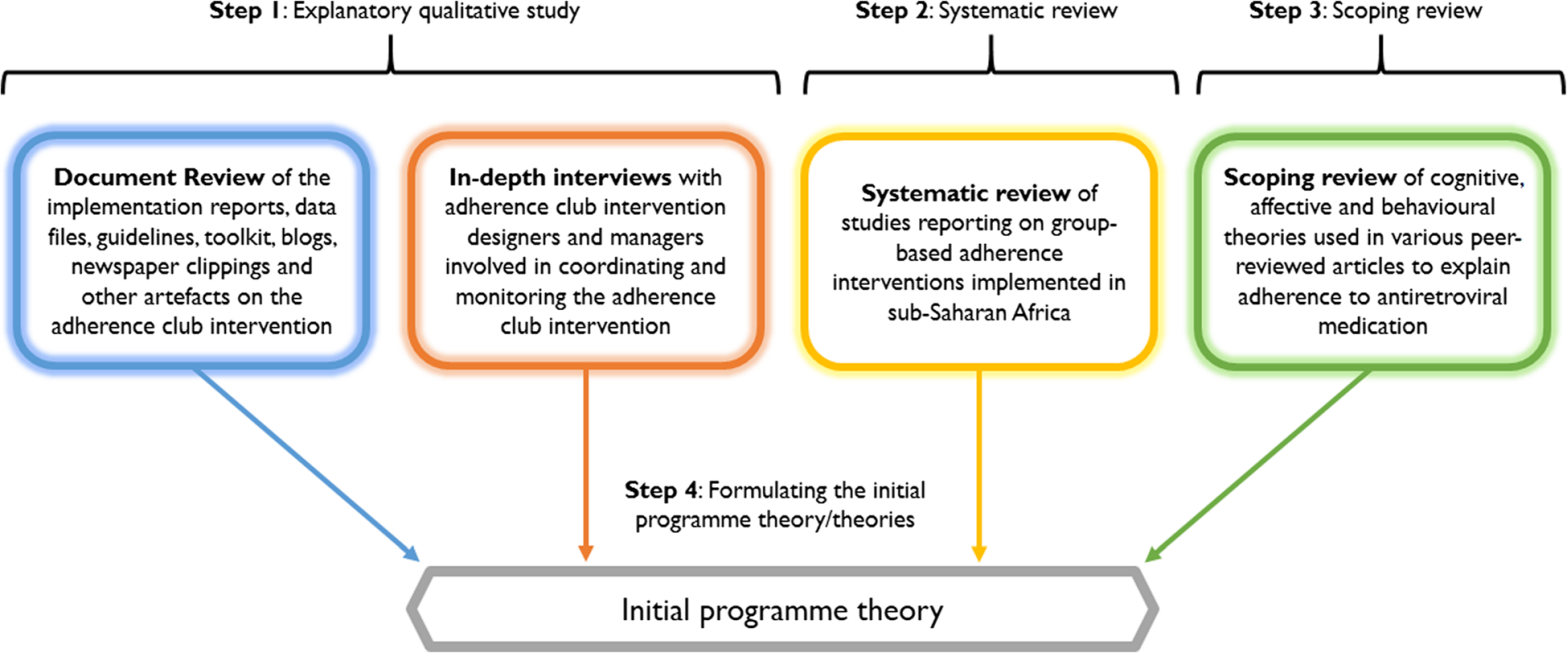
Sources of information for eliciting the initial programme theory

To obtain information on the relevant intervention, actors, mechanisms, outcomes and contexts, realist evaluators typically review programme documentation (policy documents, implementation reports, programme descriptions, etc.), interviews or discuss with various programme stakeholders, and/or draw on a systematic review of previous research and evaluation literature. These elements are then conceptually aligned using the ICAMO configurational tool, following the generative model to obtain the programme theory (theories). Figure 3 shows the various methods applied to obtain relevant information for the formation of the initial programme theory of the adherence club intervention (the process is described in more details in the results section).

For each of these steps, we used specific methods and sources of information. In the first step, we conducted an exploratory qualitative study, employing two methods: a document review of 12 documents on the design and implementation of the adherence club intervention, and key informant interviews with 12 purposively selected programme designers and managers [44]. The thematic content analysis was used to identify themes attributed to the programme actors, context, mechanisms, and outcomes (Table 1). A detailed description of the study design, methods used, and findings are reported elsewhere [44].

**Table 1:** Outcome, context and mechanism components obtained from the thematic analysis of the document review and in-depth interviews with programme managers and designers. Source: Mukumbang et al. [44]

Context	Mechanisms	Outcomes
Distal (Macro) Context	Provider/Management Level	Long-term Outcomes
- Monitoring and evaluation	- Buy-in	- Programme standardisation
- Higher level support	- Interaction (perceived social support)	- Retention in care and adherence to medication
- Stakeholder collaboration	- Motivation	- Healthier communities
Organisational (Meso) Context	Social mechanisms	Intermediate Outcomes
- Sustained hierarchical pressure	- Group dynamics - Social/Peer support	- Decongestion of clinic
- Human resources (staffing dynamics)	- Mutual learning (empowerment)	- Improved patient self-management
- Implementation methodology	- Bonding (sense of belonging)	
Local (Micro) Context	Patient (individual)-Level	Immediate Outcomes
- Availability of conducive space	- Encouragement	- Decreased workload for operational staff
- Programme champions	- Trust	- Decreased patient opportunity cost
- Oppressive surveillance	- Nudging	
	- Fear	
	- Motivation	

In the second step, we conducted a narrative synthesis of primary studies focusing on the mechanism(s) at work during the implementation of group-based interventions for adherence support among PLWHA on ART [46]. Twelve articles reporting primary studies on group-based models of ART care were included in the review. Evidence from these studies was analysed using thematic content analysis to identify ICAMO patterns. While all of the studies included in the review reported on outcomes and actors, the six studies that employed a quantitative study design to identify outcome patterns failed to identify aspects of the context and mechanisms. The other four studies applied qualitative approaches and two studies that applied mixed-methods studies identified some of the aspects of the context and mechanisms that could trigger the outcomes of group-based ART interventions. Nevertheless, none of the identified studies conceptualised the relationship(s) using the ICAMO heuristic tool. The entire systematic review, starting from the selection of the studies to the analysis and the findings obtained are described elsewhere [46]. Table 2 summarises the themes that were identified.

**Table 2:** Propositions obtained from the systematic review process of group-based ART models

Context	Mechanism	Outcome
- Staffing dynamics	- Active involvement in care - Perceived support from a counsellor and other health professionals. - Feeling empowered by their expanded roles	- Adherence to medication - Reduced operational staff workload
- Acceptability (buy-in) from health workers - Educational level of patients	- Understanding treatment - Trust and communication - Patient empowerment - Motivation	- Improved retention in care - Improved rate of medication adherence
- Availability of physical space for group activities - Buy-in from both operational staff and patients	- Positive peer dynamics - Sharing of experience - Bond formation among group members - Conducive environment	- Improved patient support which leads to retention in care and adherence to medication - Decongestion of clinic
- Acceptability (buy-in) from patients	- Patient satisfaction - Continuity of care	- Improved rate of medication adherence - Reduced workload for the clinicians

In step three, we conducted a scoping review to identify generative mechanisms in studies of ART adherence that employed various social, behavioural and cognitive theories to explain or predict patients’ adherence to ART. The aim of this review was to identify existing mechanisms in the theoretical knowledge of ART adherence and to verify if these mechanisms could be adapted to fit in the explanatory framework of the adherence club programme [47]. Twenty-six articles were included through searching five databases (PubMed, Ebscohost, CINAHL, PsycARTICLES and Google Scholar) using keywords and manual search of citations from the reference list of the studies identified from the electronic databases. Three theories (Information-Motivation-Behaviour (IMB), Social Action Theory (SAT) and Health Behaviour Model (HBM)) were of potential relevance to explaining the ART adherence through generative mechanisms and the influence of context of action. Six salient constructs were identified as candidate mechanisms to explain adherence behaviour of patients toward ART: motivation, self-efficacy, perceived social support, empowerment, perceived threat, and perceived benefits and barriers. The approach, methods and findings of this scoping review have been reported elsewhere [47].

### Data analysis/synthesis (theory gleaning)

#### Collating findings into an initial programme theory

**Fig. 4:**
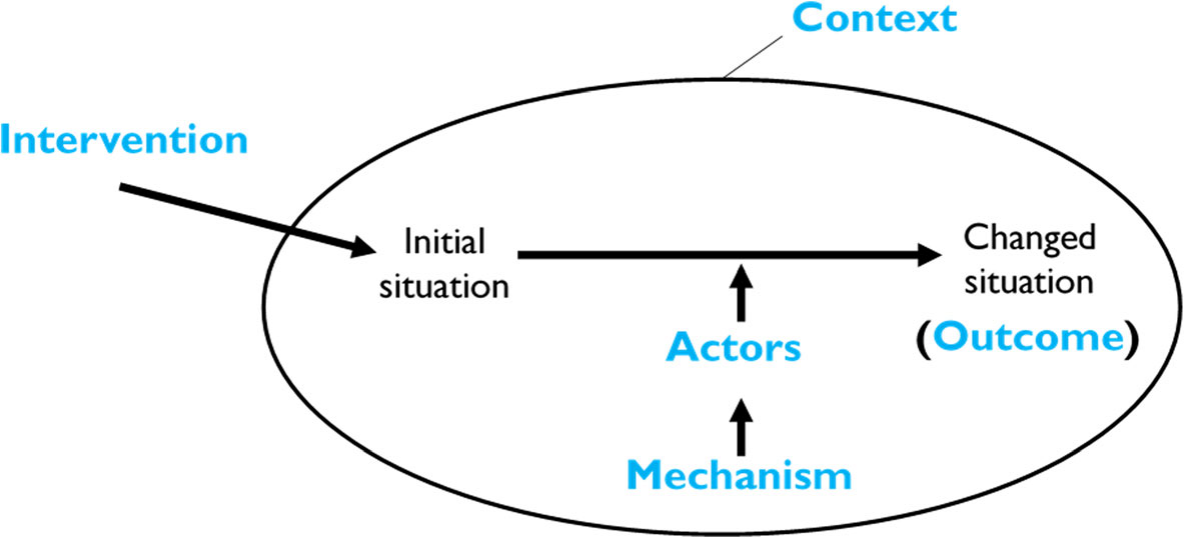
The Intervention-Context-Actor-Mechanism-Outcome heuristic tool

After identifying the various aspects of the relevant actor, context, mechanism and outcome components (steps 1–3), we used the ICAMO configuration (Fig. 4) as a heuristic to formulate the initial programme theory [23, 24].

Based on the ICAMO heuristic tool, we applied the process of configuration mapping [48] – an approach to causality, whereby, outcomes are considered to follow from the alignment of a specific combination of attributes [20]. The configuration process was achieved through the logic of *retroduction.* By applying retroduction, the realist researcher moves from the description and analysis of concrete phenomena (usually obtained from actors by interview) to reconstructing the basic conditions (theory) for these phenomena [49].

**Fig. 5:**
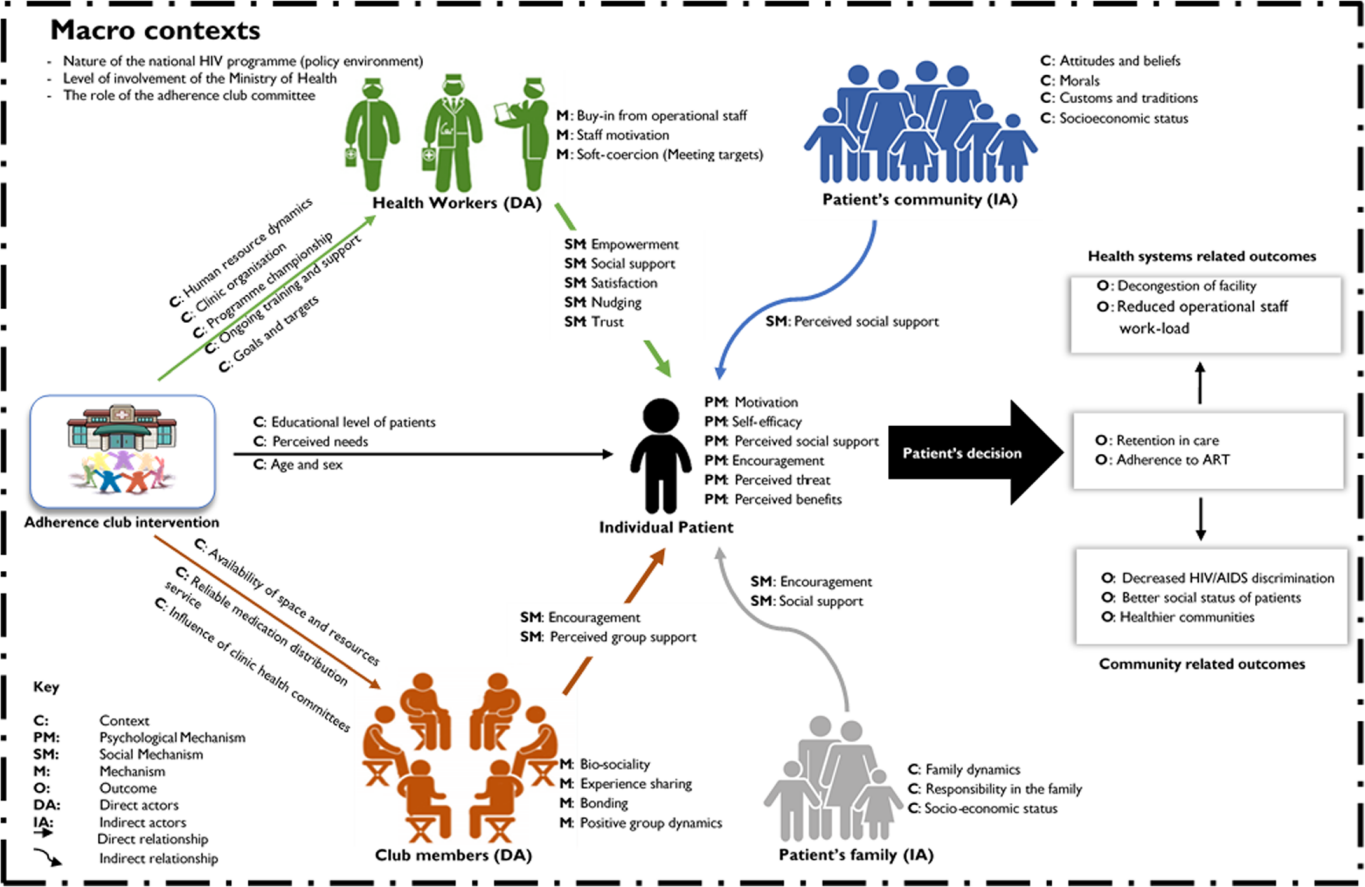
An ICAMO representation of the adherence club intervention programme theory

Following the logic of retroduction, first, we identified mechanisms that were related to the different actors (patients, health professionals) and the different outcomes that these mechanisms are likely to perpetuate. Second, we examined the context conditions that were associated with the mechanisms identified as informed by the data. Finally, to elicit the initial programme theory based on our data, we found it useful to distinguish between different sub-elements of the adherence club programme, including the individual-level, interpersonal-level, group-level, family-level and community-level. Figure 5 represents the configuration map developed through this process.

#### Formulating the testable hypotheses

The testable hypotheses were formulated by translating the ICAMO configurations using the “*if*…, *then*…, *because …*” statement [32, 48]. Through this exercise, we identified two alternative programme theories.

## Results

In the result section, we first describe that adherence club intervention casting light on the various modalities that the intervention offers, how the intervention is implemented and how, why and who executes what aspects of the intervention. This is an important part of eliciting a programme theory in the realist logic because it is the first component of the ICAMO heuristic tool and provides valuation information on context and actors. After describing the adherence club intervention, we presented the initial programme theories that were formulated through the elicitation process.

### The intervention: The adherence club programme

#### Objectives of the adherence club programme

The adherence club programme was designed to shift the majority of consultations and ART collections for stable patients to ‘clubs’ organised and facilitated by facility counsellors or peer educators [50]. In so doing, it strives (a) to retain patients – reduce the number of patients lost to follow-up – in ART care by providing a more efficient way to manage stable patients; (b) to achieve and maintain good long-term adherence in PLWHA on ART treatment by maintaining a good quality of care – creating an environment for more convenient clinical visits that accommodate their lifestyle needs; and (c) to decongest the health facility – fewer patients using the mainstream ART scheme – through group sessions that are facilitated by trained non-clinical staff [51].

#### Eligibility to the adherence club

An adherence club is made of 25–30 ‘stable’ patients on ART. Evidence from the document review shows that although patients are encouraged to request their admission into the clubs, it is clinicians who determine whether a patient qualifies for admission. Eligibility is centred on the patient being ‘stable’ – clinically well and adhering to treatment [12]. The patients can be recruited if they are 18 years or more, weigh more than 40 kg, and have been on the same ART regimen (1 or 2) for at least 12 months. Clinically, the two most recent consecutive viral loads of the patient should be undetectable (< 400 copies/mL) with a CD4 count of more than 200 cells/mm^3^ in the last 6 months [50, 52]. In addition, the patients must have a reliable clinical attendance record as evidenced by attendance card, and no medical condition requiring regular clinical consultations including tuberculosis (TB).

Club membership can be terminated if the patient has a viral load above 400 copies/mL or significantly abnormally low CD4 count < 200 cells/mm^3^, and when he/she develops an active TB infection, switches ART regimen for clinical reasons, becomes pregnant (for female patients), or develops any condition that requires frequent clinical follow-up [8]. In addition, when a patient fails to regularly attend mandatory club sessions or when he/she fails to send a ‘treatment buddy’ to collect their medication from the club facilitator or club nurse within 5 days (grace period), he/she is returned to the main clinic for care [53].

In practice, more considerations are taken into account when it comes to allowing patients in an adherence club. For instance, if a patient in the club develops a chronic co-morbidity, such as hypertension and diabetes, that is well-controlled and if the patient is ‘stable’ as per the clinician’s assessment, he/she then will still be eligible for a club on condition he/she fulfils the “HIV-related” criteria. In a similar manner, some facilities choose to keep pregnant women in the club as long as their viral load is undetectable, in which case their antenatal visits are managed by the staff responsible for antenatal care separately. However, in some settings, pregnant women are placed out of clubs and are only allowed to re-enter after pregnancy, particularly where mother and baby will be managed as a pair in the post-natal period or if antenatal care is delivered at a facility other than the facility rendering club services.

#### Management structure and running of the club activities

Running the adherence club requires a club team. Ideally, the club should be run by a five-member team: a club manager, a club facilitator, a professional nurse, a pharmacist and a data capture clerk [50]. The duties and responsibilities of each of these staff are outlined in Table 3 below.

**Table 3:** Personnel required to operate the adherence club and their responsibilities

Cadre category	Personnel type	Responsibility
Club Manager	• Medical doctor • Professional nurse	• Ensure that the Standard Operating Procedures are adhered to • Schedule annual return dates for club visits • Perform six monthly scripting of club patients • Monitor outcomes
Club Nurse	• Professional nurse	• Responsible for seeing patients who present with unexpected weight-loss and/or symptomatic for opportunistic infections and or adverse drug reactions • Collects blood for annual viral load and CD4 count screenings
Club Facilitator	• Clinic counsellor • Community health worker	• Preparing and running the club sessions • Making sure pre-pack medications are available and distributing them • Filling in the club register • Giving peer education and counselling
Pharmacist	• Pharmacist • Pharmacy assistant	• Ensures scripts are submitted and pre-packs are received and correct • Dispensing the ARVs and packaging the medication for the clubs (Chronic Dispensing Unit)
Data capturer	• Data capturer	• Captures the information on the club activities including the visit, the weight and any results entered.
‘Expert’ patient	• Patient	• Could be involved in medication distribution • Could also contribute to group education

#### Activities of the adherence club

The observations showed that facility-based adherence clubs take place in available spaces or rooms, such as the clinic boardroom, makeshift or purpose-built buildings. During the introductory visit to the club, patients are provided with the rules governing the adherence club and its activities. Patients are considered enrolled in the club after they have attended their first club meeting and have picked up their pre-packed medication. According to the document review and as confirmed by our observations, if a patient is unable to attend a club appointment for any reason, they are entitled to send someone known as a ‘treatment buddy’ – usually a family member, friend or companion who supports an ART patient on treatment – to collect their treatment from their adherence club [17].

**Fig. 6:**
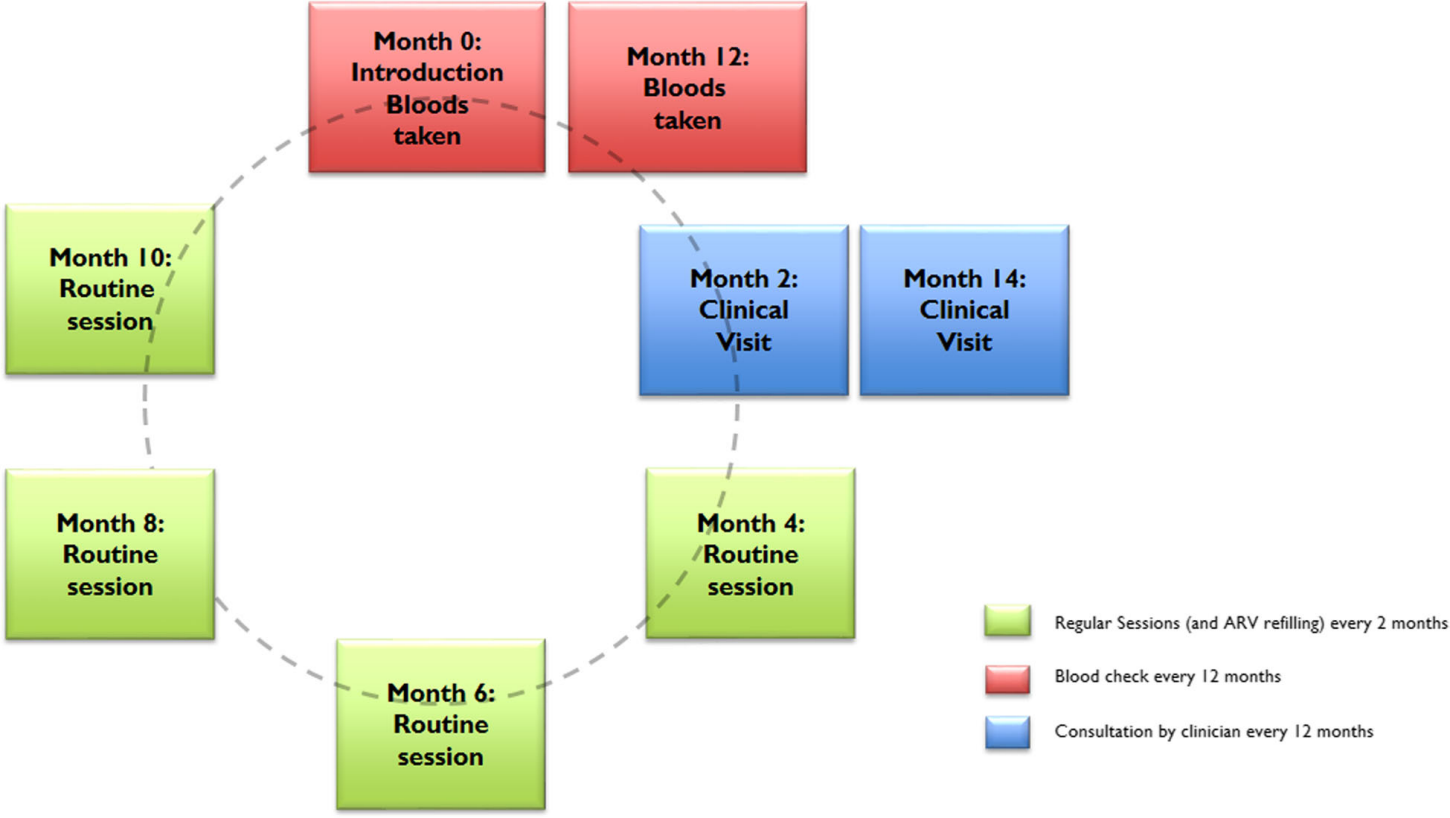
Standard annual attendance schedule of the adherence club programme

The activities of the adherence club are organised bi-monthly. A regular club session lasts approximately one to one and a half hours [50]. On days when blood tests are carried out for routine adherence monitoring through CD4 count and viral load, the sessions take much longer. Once a year, club members attend a regular clinic. Table 4 and Fig. 6 below show a standard attendance schedule of the adherence club programme.

**Table 4:** An example of adherence club annual session’s schedule [50]

Type of club visit	Activities	Script and CDU visits
	Recruitment + clinician scripting for 3 months	1 month supply by pharmacy
Enrolment visit	Scripting for 6 months	1 × 2 months pre-packed
Routine visit		1 × 2 months pre-packed
Blood visit	Bloods taken	2 × 2 months pre-packed
Clinical visit	Clinical consultation + re-scripting for 6 months	3 × 2 months pre-packed
Routine visit		1 × 2 months pre-packed
Routine visit		2 × 2 months pre-packed
Re-scripting visit	Re-scripting	4 × 2 months pre-packed

Note: The cycle repeats from month 12

Based on our observations and as reported in some of the programme documents, during a standard club session (medication pick-up), the patients arrive in the morning and hand their clinic cards to the clinic facilitator. At each visit, club members are clinically assessed (weight and symptom screen) and participate in a group adherence support and/or education activity. They are then issued with 2 months’ pre-dispensed medication. Next, the club facilitator gives health education talks to the assembled group on relevant topics. These topics include challenges that patients face in taking their medication. Sometimes a patient is asked to give a talk. The club facilitators do not have the licence to *dispense* medication; therefore, they collect the pre-packaged medication (labelled for each patient and supplied by the Chronic Dispensing Unit) and *distribute* them to the patient (or treatment ‘buddy’). The drug distribution task can also be done by an ‘expert’ patient.

**Fig. 7:**
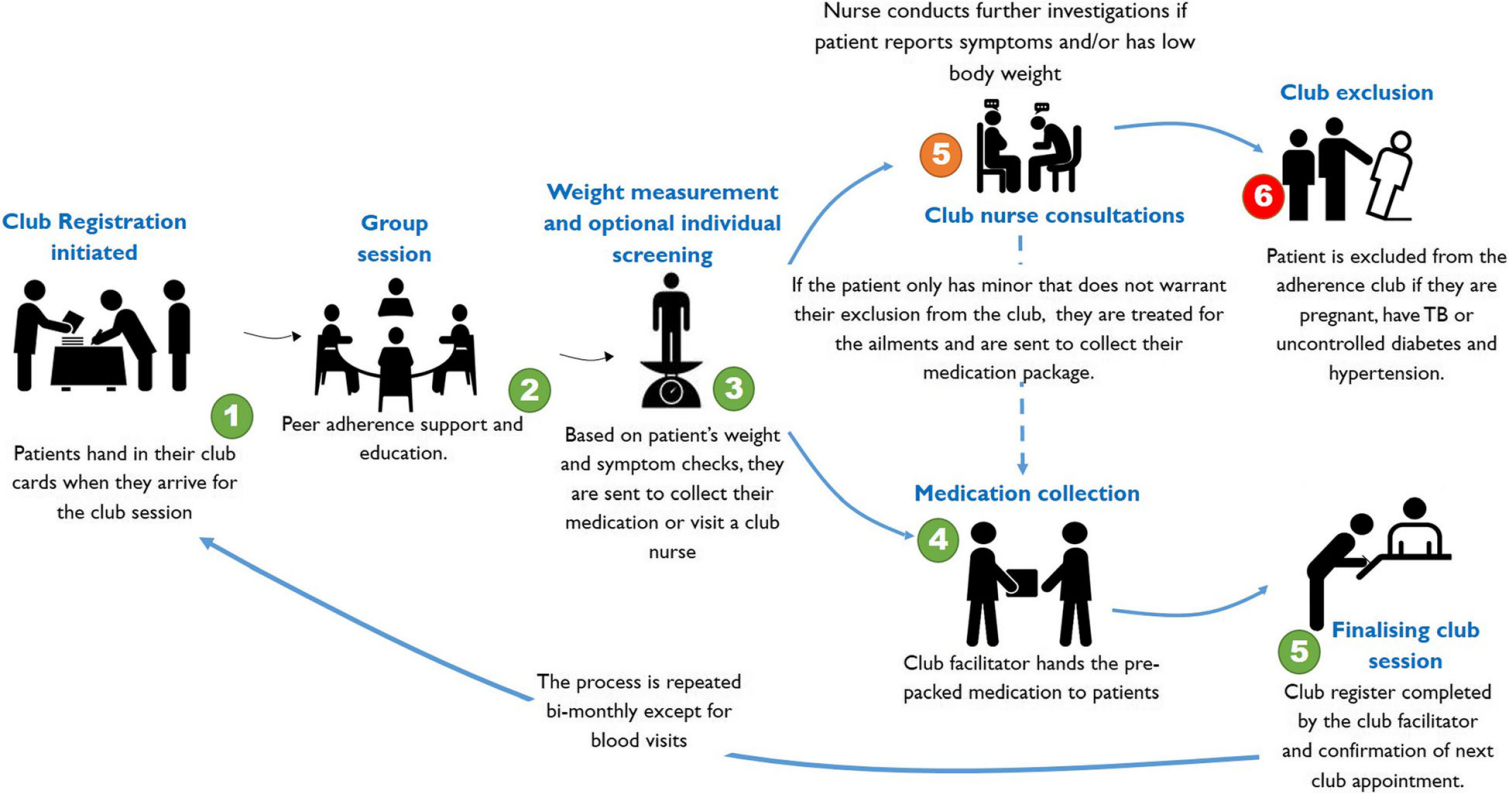
A pictorial representation of a typical facility-based adherence club session

According to the adherence club guidelines [51] and adherence club toolkit [50], any patient reporting or presenting with symptoms suggesting illness, adverse drug effects or weight loss are referred by the club facilitator to the club nurse for further consultations. Based on the outcome of the consultation, they are either sent to collect their medication from the club facilitator (minor ailments and medication side effects) or removed from the club (uncontrolled comorbidity like diabetes or hypertension). Figure 7 below outlines the activities of a standard adherence club session.

Based on the adherence club toolkit [50] and as confirmed by our observations, on the appointment days for blood checks and annual clinic visits, the patient attends in person. If a patient sends a buddy on a blood-taking or a clinical visit day or for the second time in succession, the buddy is told to inform the client that they should visit the club’s manager at the facility within five working days [51].

Based on the adherence club guidelines [51], when a club patient has not sent a ‘buddy’ to collect his/her medication and does not come to the facility within a ‘grace’ period of five working days, he/she will be referred to the club’s manager. The guideline recommends that at the end of the ‘grace’ period, the club facilitator should record the appropriate outcome of the patients who did not attend the group session after efforts to contact the patient has been made. Our observation showed that the grace period is sometimes longer than 5 days as the operational staffs tend to be more lenient. The club manager informs the patient that he/she can no longer attend the club and must return to mainstream care.

Where a patient has been returned to mainstream care for clinical reasons, the club clinician can set the conditions for the patient’s re-enrolment in the club system. In such case, the patient is considered a new club patient from a monitoring and evaluation perspective [53]. Relevant information in the club register is transferred to the facility ARV register (paper or electronic) by the clinic data clerk. Information, such as the CD4 count and annual viral load tests results, is recorded in the club register by the club facilitator and transferred to the electronic register by the data clerk.

#### Comparisons and effectiveness of the adherence club to standard clinic care

Based on structured observation of the adherence club in six health care facilities, we identified some critical differences between the adherence club model of the ART care and the standard clinic ART care. These differences are outlined in Table 5.

**Table 5:** A comparison of the adherence club intervention and the standard clinic ART service

Nature of service	Standard clinic ART care	Adherence club
Reception	Patients queue at the waiting area to be seen by a clinician. The waiting times at larger facilities can be up to 4 h.	Patients have an area reserved for them with a club facilitator at their disposal. They have scheduled times when the adherence club session starts.
Drug Dispensation	Medications are provided by the clinic pharmacy, after the consultation with the clinician. The patient is expected to queue at the pharmacy waiting area for their medication to be served. Patients receive one month’s supply of medication and possible two-month’s supply when the patient shows positive signs of adherence.	Medication is pre-packed by a central packaging and distribution centre, the Chronic Dispensing Unit, and distributed during the club session by the club facilitator. Patients receive two month’s supply of medication. They can be given up to four month’s supply of medication during festive periods.
Blood sample collection	Patients queue in front of the preparation room to be seen by a professional nurse so that blood can be drawn for routine CD4 and viral load measurements	Patients in the adherence club have a professional nurse allocated to them, who prepares their laboratory forms and collects their blood samples at the set time. Members do not have to wait.
Attendance Frequency	For each appointment, the patient is expected to be seen by a clinician for routine consultation and then by a lay counsellor for their drug accountability assessment and counselling. Patients are thus expected to attend in person at all times.	Most of the activities at the adherence clubs are conducted by the lay counsellor and the patient is only expected to consult a clinician once a year except when the patient develops any opportunistic infection. Patients can send a ‘buddy’ to collect their medication and can only show up when it is time for their blood to be collected.
Accountability	There is less accountability for these patients as they are not allocated to particular lay counsellors	Better accountability and follow-up from the club facilitator as they feel responsible for the smooth running of their clubs and the patients in their clubs.

Both the facility-based and community-based adherence club models have shown better results and effectiveness in retaining patients in care and improving adherence to medication compared to the regular clinic services under pilot conditions [11, 12]. An evaluation of the adherence club intervention under implementation conditions confirms the findings of the pilot studies [54]. Recent evidence shows that patients receiving care in the adherence care model show more satisfaction with ART care compared to those who receive care from the standard ART scheme [55]. Evidence also shows that the adherence club model of ART care is cost-effective compared to standard care [56].

### The programme theory

Based on the above process described to elicit the initial programme theory of the adherence club programme, two salient programme theories were identified.

#### Programme theory 1

From our analysis, we identified quick access to medication at the convenience of the patient with reduced frequency (every two months), regular counselling and education sessions, and quick access to a clinician when required as central elements of the adherence club intervention. The context includes the availability of resources, programme buy-in from the operational staff and the club and clinic organisational culture and activities. Important actor-related elements include the educational level of patients, the willingness to be included in the intervention, and age and sex of the patient. Self-efficacy, motivation, perceived social support, encouragement, perceived threat and perceived benefits emerged as individual-level mechanisms. Grouping patients in the adherence club may trigger other mechanisms operating at the group level such as experience sharing, bonding and group dynamics. Programme Theory 1 is succinctly expressed in the following quotes:

***“IF*** *adult (18+years) clinically stable patients with evidence of good clinic attendance are group-managed and receive quick symptom checks, quick access to medication, consistent counselling and social support from the peer counsellor,*

***THEN*** *these patients are likely to adhere to medication and remain in care,*

***BECAUSE*** *they develop a group identity, which improves their perceived social support, satisfaction and trust, and acquire knowledge, which helps them to understand their perceived benefits and improves their self-efficacy.*

*As a result, they become empowered and motivated, thus, more likely to remain in care and adhere to the treatment.* ***”***

#### Programme theory 2

The second programme theory is an alternative theory to the first and suggests that the patients are being nudged, pressured, or coerced to attend club activities. In other words, an external force prompts the patients to attend the club activities rather than their own volition, control or willingness. The nudging process stems from the desire of the health system managers to decongest the health facilities by keeping the patients in club care. Thus, goals and targets are set at the provincial level and imposed on the facility to enrol more patients into the adherence club programme and maintain them within the club system. The promise of being returned to the main treatment stream if the patient misses a club appointment and did not send a treatment buddy is implemented by the operational staff to ensure that patients remain in care. This promise translates into perceived threat (fear of losing the privileges patients enjoy as adherence club members). Programme theory 2 of the adherence club intervention is summarised in the quote below.

***“IF*** *operational staff receive goals and targets set to continuously enrol patients in the adherence club and strictly monitor their participation through strict standard operating practices (The promise of exclusion in the event of missed appointment and poor adherence outcomes),*

***THEN*** *patients are likely to adhere to medication and remain in care,*

***BECAUSE*** *they fear (perceived threat) losing the benefits (easy access to medication, peer support, reduced waiting times, and two-month ART collection) of the club system and they are coerced through adhesive club rules.*

*As a result, they become nudged to remain in care and adhere to the treatment, which might decongest the health facility.* ***”***

## Discussion

We elicited the initial programme theory by gleaning information from various sources. Constructing and refining the initial programme theory required us to explore the assumptions and perspectives of the programme managers and designers on how the adherence club could work. Consolidating the initial programme theory, which we elicited from the document review and the programme designers, required that we explored evidence on how similar interventions worked (or not) in other contexts and to explore cognitive and behavioural theories that have been applied to explain adherence to ART behaviours. We integrated and conceptualised the evidence from these sources using the ICAMO configuration to formulate the initial programme theories. Based on our configurational mapping, two programme theories, each offering an alternative explanation of how, why and in what circumstance the adherence club works, emerged.

According to Pawson and Manzano-Santaella [57], “programmes never offer up a single theory”. This is because of the multiple mechanisms – a proliferation of ideas within a programme, creating different resources that trigger different reactions among the actors [57]. In a similar manner, there are the individual-level, institutional-level and structural features that modify the action of the assorted mechanisms provided through the adherence club intervention. This indicates that there are multiple context conditions involved in modifying how the intervention plays out, which presupposes multiple potential outcomes. The theories obtained in this study will be tested during the next phase of our study. The initial programme theories will guide the design and data collection methods for that empirical work [25].

Pawson and Tilley [20] suggested that while eliciting a programme theory based on the realist logic, the findings (theories) obtained should have three main characteristics: ‘configurational’, ‘middle-range’ and ‘adjudicationist’. By configurational, the authors implied that the theories should be able to show how combinations of programme-related attributes need to be in place for a programme to be effective. With regard to theories being at the ‘middle range’, they said the theories should have the potential for transferability on the basis that they are imbued with concepts that “link to other programme theories and thus rest on further bodies of findings.” Finally, with regard to being adjudicationist, the findings obtained should provide alternative explanations to the fore to sift and sort them. Our programme theories possess these three characteristics.

In accordance to the three main characteristics of a realist programme theory, we applied the retroduction logic to configure the elements of the realist heuristic tool (configurational mapping) – assuming the outcomes to follow from the alignment of mechanisms fired in particular contexts – to construct the programme theory. For the middle range characteristic, we considered the explanatory potential of other ART adherence theories and models that have been previously developed, in addition, we explored how and why other group-based adherence-enhancing models work (or not). Finally, concerning adjudication, our findings present two potential rival theories that could be tested and refined accordingly.

It was found by Marchal and colleagues [58] that in attempting to specify the context-mechanism-outcome configurations while formulating realist programme theories, some authors fail to demonstrate the explanatory nature of the realist logic. Authors often come up with exhaustive fragmented ‘catalogues’ of plausible contexts, followed by other lists of mechanisms and another list of outcomes as opposed to properly structured and interconnected relationships between programme context, its mechanism and outcomes. While formalising the testable hypotheses, we used the retroductive inferencing logic to configure the relationship between these elements, and the “if…, then…, because…” statements to translate the ICAMO configurations into testable theory. This approach limits the chances of simply cataloguing the elements of the intervention, context, actor, mechanisms and outcomes.

We found that the configurational logic of the realist analysis was strengthened by including a description of the ‘Intervention’ and of the ‘Actors’ in the CMO heuristic [23, 24]. Dalkin et al. [59] and Jagosh et al. [28] also proposed modifications to the CMO heuristic. Dalkin et al. [59] suggest the importance of conceptualising mechanisms as operating on a continuum, rather than as an ‘on/off’ switch. They argue that this conceptualisation can have more explanatory value in understanding how interventions work. Jagosh et al. [28] suggested that context-mechanism-outcome configurations could be linked to each other in a ‘ripple effect’ type pattern. We found that adopting the ICAMO configuration stimulates the realist analyst to identify and distinguish the configuration for each group of actors as a function of the different modalities of intervention implementation [60].

### Study limitations

The identified limitation to studies of this nature relates to the fact that the process of making the associations of the various elements in the configurational mapping depends largely on the judgement of the researcher(s). This process could be, therefore, subjective based on the interpretation that the researcher applies to the available data. This limitation was minimised by the use of more than one researcher in the process of making the analysis and connections and by presenting the configurational map at a journal club for comments. Comments received at the journal club were also applied to refine the final configuration map obtained.

Our study of the adherence club intervention was limited to the South African context, specifically to the Western Cape Province. This is because, it is in this region that the adherence club intervention was designed, developed and first implemented. Working within this context provides us with rich information pertaining to the history and developmental changes that the programme underwent to its present form.

## Conclusion

Although there is a wealth of evidence suggesting that adherence clubs have the potential to improve access to ART, retention in care and adherence to medication, challenges remain regarding their conceptualisation, scalling-up and implementation [61]. Our theory-based understanding, using the realist approach and retroduction, could provide valuable implementation insights of how the different components of the adherence clubs work (or not) and under what conditions. To the best of our knowledge, there is a death in the use of realist evaluation and other theory-driven approaches to evaluate HIV-related programmes. This article was meant to encourage the use of theory-driven evaluation approaches such as realist evaluation approach.

In this paper, we described the process of eliciting the initial programme theory of the adherence club intervention. Based on our analysis, we identified two testable hypotheses for the adherence club intervention. The first theory supposes that patients become encouraged, empowered and motivated through the adherence club intervention to remain in care and adhere to the treatment [62]. The second theory suggests that stable patients on ART are being nudged to remain in care and adhere to the treatment with the goal to decongest the primary health care facilities [62]. These programme theories were the starting point of an evaluation of the adherence club programme. We tested the two hypotheses elicited in three contrastive sites to obtain a refined theory [24].

## Additional files

Additional file 1: A table of documents included in the document review. (DOCX 15 kb)

Additional file 2: Nonparticipant observation guide. (DOCX 14 kb)

## Abbreviations

AIDS: Acquired Immune Deficiency Syndrome
ART: Antiretroviral therapy
CMO: Context-Mechanism-Outcome
CoCT DoH: Cape Town Municipality City Health department
DoH: Department of Health
HBM: Health Belief Model
HIV: Human Immuno-virus
ICAMO: Intervention-Context-Actor-Mechanism-Outcome
IHI: Institute for Healthcare Improvement
IMB: Information-Motivation-Behaviour
MSF: Médecins Sans Frontière
PLWHA: People Living with HIV and AIDS
SAT: Social Action Theory
TAC: Treatment Action Campaign
WC: Western Cape
