# Supplementary material for: A realist approach to eliciting the initial programme theory of the antiretroviral treatment adherence club intervention in the Western Cape Province, South Africa
Source: BMC Med Res Methodol. 2018 May 25;18:47. doi: 10.1186/s12874-018-0503-0 (PMC5970495; doi:10.1186/s12874-018-0503-0)
Supplement: Supplementary file 1 — A table of documents included in the document review. (DOCX 15 kb) [file 12874_2018_503_MOESM1_ESM.docx]

| Title | Author/year | Document Type | | Description |
| --- | --- | --- | --- | --- |
| The adherence club toolkit | MSF/WCPG (2013) | Toolkit | This toolkit provides a detailed account on how to establish clubs, the ART club staff organogram, lessons learned through the Khayelitsha implementation experience and tools utilised in the ART club model. | |
| Adherence club register | MSF/WCPG | Adherence Club register | This document is the adherence club register that the club facilitator fills in during every club meeting. | |
| ART adherence clubs: A long-term retention strategy for clinically stable patients receiving antiretroviral therapy | Wilkinson LS (2013) | Journal Article | This article describes the adherence club programme (structure and function). It also elaborates on the implementation strategy that was employed and provides the experiences from the implementation of the adherence clubs in Khayelitsha. | |
| Treating Millions for HIV — The Adherence Clubs of Khayelitsha | Champion EW (2015) | Journal Article | This article describes the experiences of the author as he investigated the functioning of a community-based adherence club in the home of a club member. He also reports on an interview that he had with the coordinator of the adherence club programme for MSF. | |
| Out-of-clinic adherence club for delivery of ARVs shows better retention than standard of care | Odendal L (2012) | Online-news Article | This article discusses the advantages of the adherence club programme over the standard clinic care with regard to retention in care and adherence. It elaborates on a comparative study that was conducted to investigate the effectiveness of the adherence club, the findings of the study and the implications. | |
| Reaching closer to home: Progress implementing community-based and other adherence strategies supporting people on HIV treatment | SAMU & MSF (2013) | Report | This document describes the progress that has been made in implementing community-based models of ART care since the release of the report “Closer to Home” by UNAIDS and MSF in July 2012. | |
| MSF again paves the way with ART | Bateman C (2013) | Journal Article | This article provides a general description of the adherence club, emphasising the superiority of the adherence club model of care over the standard clinic care. The article states some conditions that are necessary for the adherence club initiative to be successful. The article ends by providing a doctor’s perspective on the adherence clubs. | |
| Clubbing together for treatment | Health-e News (2012) | Online-Health News Article | This article describes the adherence club intervention and its role in reducing patient loads (ART initiation). It discusses the effectiveness of the adherence club, and how this could be replicated in other areas. | |
| Western Cape ART-Adherence Treatment Clubs and Preventative Therapy for New-borns | WCG (2014) | Online-news Article | This news article was written on the inauguration of the World AIDS day on December 2014. It describes the progress that has been made on the retention in care of PLWHA since the inception of the adherence clubs. It also reports on the progress made in the implementation of the adherence club in the Western Cape Province. | |
| Guidelines for ART clubs | Western Cape Government (2015) | Standard Operating Practice (SOP) of the adherence club | This document describes the standard operating practices of the adherence club. It starts by describing the aims and the objectives of the adherence club, outlines the requirements to establish the adherence club, the organisation and running, the pharmacy requirements for the scripting and dispensing medication to ART patients and finally, the scripting schedule of the ART medication. | |
| Implementation scale up of the Adherence Club model of care to >30,000 stable ART patients in the Cape Metro, South Africa 2011- 2015 | Wilkinson, L. et al. (2015) | Conference presentation and Journal article | This presentation focuses on the nature of the adherence club and its impact on the retention in care rates of PLWHA. It also describes the possible different types of adaptation of the programme. | |
| Closer to home: Delivering antiretroviral treatment therapy in the community: Experiences from four countries in Southern Africa | MSF/UNAIDS (2012) | Report | This paper describes the implementation and the results of community-based methods of delivering ART in communities in four Southern African countries. | |

Table of documents included in the document review
