# Supplementary material for: A realist approach to eliciting the initial programme theory of the antiretroviral treatment adherence club intervention in the Western Cape Province, South Africa
Source: BMC Med Res Methodol. 2018 May 25;18:47. doi: 10.1186/s12874-018-0503-0 (PMC5970495; doi:10.1186/s12874-018-0503-0)
Supplement: Supplementary file 2 — Nonparticipant observation guide. (DOCX 14 kb) [file 12874_2018_503_MOESM2_ESM.docx]

**Structural nonparticipant observations Adherence Club Observation Guide**

**Nonparticipant observations**: The aim of conducting the non-participant observations is to

- describe the **Intervention –** operational implementation of adherence club (how the various activities of the adherence club are conducted),
- the interactions between the club facilitator and club members and between the club members (**actors**)
- the **context** within the which the participants of the adherence club interact and
- The immediate outcome of the adherence club intervention

**Intervention**:

Observe if the planned activities of the adherence club are followed as planned or outlined in the club design.

Was the time respected? This is the starting and finishing times.

How much innovation takes place within the intervention itself?

Do the club members take part in running parts of the adherence club activities such as distribution of medication, sharing of experiences and giving health educational talks and advise?

**Context:**

Is there a designated space for the adherence club activities and what prior preparations are made? How is it set up physically? Draw the site or take a photo (ethics permitting)

Is there any form of information sharing among the club members? Whether formal or informal?

Is there is camaraderie among the club members?

How is the overall vibe in the adherence club? Tense? Friendly? Cordial?

**Actors**:

The interactions between the club members and the club facilitator. What can be observed in the relationships that they share and how they interact?

The interactions between the group members: Do the group members interact with each other. How can their interactions be described?

**Outcomes**:

The attendance rate of the club.

What was the countenance of the club attendees?

What were their reactions at the end of the club session?
